# Supplementary material for: Characteristics of perceived effective telesupervision practices: A case study of supervisees and supervisors
Source: PLoS One. 2023 Jul 11;18(7):e0288314. doi: 10.1371/journal.pone.0288314 (PMC10335680; doi:10.1371/journal.pone.0288314)
Supplement: S1 Appendix — (DOCX) [file pone.0288314.s001.docx]

**S1 Appendix - Interview Guide**

1. How would you describe a successful telesupervision arrangement?
2. Please explain what lead you to conclude that your switch to telesupervision arrangement is working at least as effectively as your previous face-to-face clinical supervision arrangement?

- What are the things that are working well in the telesupervision arrangement?
- What things, if any, can be improved?

1. What changes did you have to make to your clinical supervision arrangement, if any, before switching to the telesupervision arrangement?

- Has the way you provide feedback and its content changed because of technology? If so how?

1. Can you explain how the switch over to telesupervision has influenced your clinical supervision content and activities undertaken within the session?

- What changes have been made to replace face-to-face hands-on client interactions/ skills practice etc.? How is this still meeting the supervision goals?
- Level of confidence in undertaking these activities using technology

1. Can you explain how the use of technology has impacted the supervisory relationship you share with your supervisor/supervisee?

- Give examples
- How has the supervisory relationship in telesupervision been evaluated?

1. What recommendations do you have for others setting up telesupervision arrangements, so that they can achieve comparable outcomes to face-to-face supervision arrangements?
