# Supplementary material for: Characteristics of perceived effective telesupervision practices: A case study of supervisees and supervisors
Source: PLoS One. 2023 Jul 11;18(7):e0288314. doi: 10.1371/journal.pone.0288314 (PMC10335680; doi:10.1371/journal.pone.0288314)
Supplement: Attachment — Submitted filename: Response letter_22May23.docx [file pone.0288314.s002.docx]

**Characteristics of perceived effective telesupervision practices: A case study of supervisees and supervisors**

**Response to reviewers’ comments**

We thank the academic editor and reviewers for providing valuable feedback on our manuscript (PONE-D-22-33318). Please find below a table of changes we have made to the manuscript following the review.

|  | **Academic editor comments** | **Authors’ response** |
| --- | --- | --- |
| 1 | Thank you for stating the following financial disclosure:  “Declaration of funding  This manuscript is a part of Dr Priya Martin's postdoctoral fellowship which was funded through an Advance Queensland Industry Research Fellowship Grant by the Queensland Government Department of Tourism, Innovation and Sport; and co-funded by Darling Downs Health, Southern Queensland Rural Health, the University of Queensland Rural Clinical School, and the University of South Australia. Grant number - AQIRF115-2020-CV.”  Please state what role the funders took in the study.  If the funders had no role, please state: ""The funders had no role in study design, data collection and analysis, decision to publish, or preparation of the manuscript.""  If this statement is not correct you must amend it as needed.  Please include this amended Role of Funder statement in your cover letter; we will change the online submission form on your behalf. | Here is the updated **Declaration of funding** statement:  This manuscript is a part of Dr Priya Martin's postdoctoral fellowship which was funded through an Advance Queensland Industry Research Fellowship Grant by the Queensland Government Department of Tourism, Innovation and Sport; and co-funded by Darling Downs Health, Southern Queensland Rural Health, the University of Queensland Rural Clinical School, and the University of South Australia. Grant number - AQIRF115-2020-CV. The funder (i.e., Queensland Government Department of Tourism, Innovation and Sport) had no role in study design, data collection and analysis, decision to publish, or preparation of the manuscript. |
| 2 | In your Data Availability statement, you have not specified where the minimal data set underlying the results described in your manuscript can be found. PLOS defines a study's minimal data set as the underlying data used to reach the conclusions drawn in the manuscript and any additional data required to replicate the reported study findings in their entirety. All PLOS journals require that the minimal data set be made fully available.  We note that you have indicated that data from this study are available upon request. PLOS only allows data to be available upon request if there are legal or ethical restrictions on sharing data publicly. | Our study of a small cohort contains potentially sensitive information on participants’ clinical supervision practices. The study is protected by ethics approval obtained from the Darling Downs Human Research Ethics Committee for multi-sites. Participant consent to share the de-idetified dataset on a public repository was not obtained at the time of the study. Therefore, any reasonable request to access this dataset needs to be directed to the Darling Downs Human Research Ethics Committee via email at [DDHHS-RESEARCH@health.qld.gov.au](mailto:DDHHS-RESEARCH@health.qld.gov.au) |
| 3 | Please review your reference list to ensure that it is complete and correct. If you have cited papers that have been retracted, please include the rationale for doing so in the manuscript text, or remove these references and replace them with relevant current references. Any changes to the reference list should be mentioned in the rebuttal letter that accompanies your revised manuscript. If you need to cite a retracted article, indicate the article’s retracted status in the References list and also include a citation and full reference for the retraction notice. | Done. |
|  | **Reviewer 1 comments** | **Authors’ response** |
| 1 | Thanks for undertaking this important work that is going to be increasingly relevant in the new normal of a post-COVID world. I agree with the authors' observation that this approach may no longer just be confined to rural areas. | Thank you for the positive feedback. |
| 2 | While the paper is generally well written I think there are a couple of areas that I think need a little more explanation:  - what are the specifics of the supervisory arrangements? For example they may be legislative (eg supervision of medical interns), educational (eg Registrars in general practice or teaching hospitals) or just good practice. Describing the context would be helpful in understanding the generalizability of the findings - Proctor's tripartite model may not be familiar to all readers so I would suggest a brief explanation of the terminology used | This has now been added (*lines 6-8*):  For several professions, clinical supervision is mandated through professional associations, registration guidelines, and/or organisational policies and directives.  This has now been added (*lines 14-18*):  Formative functions include the supervisee gaining skills and knowledge (e.g., conducting an assessment, making a splint). Normative functions include clinical governance and quality assurance (e.g., compliance with policies, guidelines, protocols). The restorative function is dedicated to supporting the supervisee’s emotional wellbeing at work. |
| 3 | Also, I note there is no mention of programs like the Remote Vocational Training Stream (<https://rvts.org.au/>) which has been using remote telesupervision for over 20 years. | Information about RVTS wasn’t included as it is a highly structured national training program of doctors completing a fellowship in General Practice. This study in contrast concerns clinical supervision of practicing healthcare workers (all allied health professionals) who have completed all training requirements and work within the public healthcare sector in Queensland. |
| 4 | The findings seem appropriate and grounded in the data, although I note there were only three sets of interviewees, which seems low. A comment on why this number was chosen, and whether data saturation was achieved may be worth considering. | This has now been further clarified in the **strengths and limitations** section (*lines 335-339*):  Recruiting healthcare workers to participate in research during this time period was challenging given the workforce issues triggered by the COVID-19 pandemic, hence this study only included three supervisor-supervisee pairs. However, this challenge was mitigated by using in-depth exploration and two sources of data to provide a thorough view, and through reaching data sufficiency. |
| 5 | The paper is generally well written and clear, although I wonder about the use of the term 'content experts' in line 111 - I think the expertise described is more process-based / educational than in the content being taught.  Finally, line 253 says 'Several healthcare workers...' - I suspect it was more than this, and these words should be revised. | The term ‘content experts’ was used as the researchers have expertise in clinical supervision training and research. This has now been clarified (*line 121*):  …both content experts in clinical supervision, with extensive experience in clinical supervision training and research, and qualitative research methods…  The word ‘several’ has now been removed (*line 263*). |
| 6 | Thank you for the opportunity to review this paper. | Thank you for reviewing our paper and providing constructive feedback. |
|  | **Reviewer 2 comments** | **Authors’ response** |
| 1 | Thank you for conducting this study. The topic is very interesting, current and useful for better understanding and utilizing telesupervision.  Below are some suggestions: | Thank you for the positive feedback. |
| 2 | In the Introduction paragraph, I suggest providing more justification for the choice of Case study method and the Proctor model for data analysis. | More information has now been added about the Proctor’s model (*lines 14-18*):  Formative functions include the supervisee gaining skills and knowledge (e.g., conducting an assessment, making a splint). Normative functions include clinical governance and quality assurance (e.g., compliance with policies, guidelines, protocols). The restorative function is dedicated to supporting the supervisee’s emotional wellbeing at work.  Justification for the case study method is located in the **Research design** section (*lines 73-78*):  An instrumental-use multiple-case method^16^ was used where particular cases are studied to gain a broader appreciation of an issue.^17^ This case study method was chosen as it focuses on generating useful and actionable findings, as well as identification of factors that explain the difference between what works and what doesn’t work, which can then be used to inform decision making and to support policy and practice.^16,18^  In addition, the study aim has been re-pharsed to make this clearer (*lines 65-66*):  Given this increasing use of telesupervision and a dearth of evidence in this area, including in-depth exploration, this current study utilised a case study approach to explore factors that influence effective telesupervision practices from a supervisor and supervisee perspective. |
| 3 | In the Materials and Methods section, I suggest providing more explanation for the choice of the overarching constructionist theoretical paradigm and the sample selection method. It is not clear how the selecting cases was conducted. Was the snowball sampling strategy used? At the end, were the 6 participants the most relevant to the issues studied? | We have detailed that a “constructionist theoretical paradigm was used to allow for co-construction of knowledge and meaning in the given context. This allowed the researchers to hear all participant views with equal weighting” (*lines 71-73*).  Sampling consisted of utilisation-focused sampling (information provided already in the **Research design** section; *lines 78-86*) and snowball sampling (*line 89*). Further information has been added about recruitment (*lines 86-89*):  Participants were recruited through promotion at the end of previous survey studies in this population, whereby they were asked to contact the first author should they be interested in participating in the case study. ^19, unpublished^ Further, a snowball sampling strategy was used additionally to recruit participants.  A section in the **Strengths and limitations** paragraph explains participant numbers recruited (*lines 335-339*):  Recruiting healthcare workers to participate in research during this time period was challenging given the workforce issues triggered by the COVID-19 pandemic, hence this study only included three supervisor-supervisee pairs. However, this challenge was mitigated by using in-depth exploration and two sources of data to provide a thorough view, and through reaching data sufficiency. |
| 4 | Lastly, in the Results section, under the category 'Right supervisor and right supervisee,' I suggest highlighting the characteristics of the supervisor that are useful in telesupervision, as the characteristics listed (such as open and honest, supportive, good at feedback provision, non-judgmental, approachable) seem general, regardless of whether the supervision is remote or face-to-face. | The peer reviewer has made the right observation. The corresponding findings section has been slightly re-worded to make this point (*line 245*): In line, with best practice in face-to-face clinical supervision…  This has then been picked up in the **discussion** section (*lines 321-325*):  It is noteworthy that characteristics of a ‘right’ supervisor and ‘right’ supervisee for telesupervision is similar to that of face-to-face supervision arrangements. Previous research has noted that while there are similarities, a higher dosage of these characteristics or traits are required in telesupervision to make up for the absence of in-person contact. ^5,28^ |

Kind regards,

Authorship team
